# Immune checkpoint inhibitor related myasthenia gravis: single center experience and systematic review of the literature

**DOI:** 10.1186/s40425-019-0774-y

**Published:** 2019-11-21

**Authors:** Houssein Safa, Daniel H Johnson, Van Anh Trinh, Theresa E Rodgers, Heather Lin, Maria E Suarez-Almazor, Faisal Fa’ak, Chantal Saberian, Cassian Yee, Michael A Davies, Sudhakar Tummala, Karin Woodman, Noha Abdel-Wahab, Adi Diab

**Affiliations:** 1 Department of Melanoma Medical Oncology, The University of Texas MD Anderson Cancer Center, Houston, TX USA; 2 Department of Biostatistics, The University of Texas MD Anderson Cancer Center, Houston, TX USA; 3 Section of Rheumatology and Clinical Immunology, Department of General Internal Medicine, The University of Texas MD Anderson Cancer Center, Houston, TX USA; 4 Department of Internal Medicine, Piedmont Athens Regional Medical Center, Athens, Georgia; 5 Department of Neuro-Oncology, The University of Texas MD Anderson Cancer Center, Houston, TX USA; 6 Department of Rheumatology and Rehabilitation, Faculty of Medicine, Assiut University Hospitals, Assiut, Egypt

**Keywords:** Myasthenia gravis, Immune checkpoint inhibitors, Nivolumab, Pembrolizumab, Ipilimumab, Immunotherapy

## Abstract

**Background:**

Myasthenia gravis (MG) is a rare but life-threatening adverse event of immune checkpoint inhibitors (ICI). Given the limited evidence, data from a large cohort of patients is needed to aid in recognition and management of this fatal complication.

**Methods:**

We reviewed our institutional databases to identify patients who had cancer and MG in the setting of ICI. We systematically reviewed the literature through August 2018 to identify all similar reported patients. We collected data on clinical and diagnostic features, management, and outcomes of these cases.

**Results:**

Sixty-five patients were identified. Median age was 73 years; 42 (65%) were males, 31 (48%) had metastatic melanoma, and 13 (20%) had a preexisting MG before ICI initiation. Most patients received anti-PD-1 (82%). Sixty-three patients (97%) developed ICI-related MG (new onset or disease flare) after a median of 4 weeks (1 to 16 weeks) of ICI initiation. Twenty-four patients (37%) experienced concurrent myositis, and respiratory failure occurred in 29 (45%). ICI was discontinued in 61 patients (97%). Death was reported in 24 patients (38%); 15 (23%) due to MG complication. A better outcome was observed in patients who received intravenous immunoglobulin (IVIG) or plasmapheresis (PLEX) as first-line therapy than in those who received steroids alone (95% vs 63% improvement of MG symptoms, *p = 0.011)*.

**Conclusions:**

MG is a life-threatening adverse event of acute onset and rapid progression after ICI initiation. Early use of IVIG or PLEX, regardless of initial symptoms severity, may lead to better outcomes than steroids alone. Our data suggest the need to reassess the current recommendations for management of ICI-related MG until prospective longitudinal studies are conducted to establish the ideal management approach for these patients.

## Introduction

Immune checkpoint inhibitors (ICI) such as cytotoxic T-cell lymphocyte associated antigen-4 (CTLA-4) and programmed cell death-1/programmed cell death-ligand 1 (PD-1/PD-L1) blocking agents are indicated as a standard of care in several cancers [1–3]. Their use is expected to expand, including in the adjuvant setting, leading to an increase in the population of cancer patients exposed to these therapies [4–8]. However, the clinical benefit of ICIs can be limited by toxicities caused by off-target inflammatory and autoimmune responses, which can be life-threatening, and may require treatment discontinuation and initiation of immunosuppressants. A challenging population is patients who have a dual diagnosis of autoimmune diseases and cancer, requiring the use of ICIs [9–11]. A recent systematic review of the literature summarized the evidence on adverse events associated with ICI use in patients with preexisting autoimmune diseases and found that most of these patients (75%) are susceptible to develop flare of the underlying autoimmunity (50%), and/or new onset immune-related adverse events (irAEs) (34%) [9]. Although adverse events improved in most patients (90%), some (17%) required permanent ICI discontinuation or remained at risk of serious complications including fatality (4%).

ICI-related neurologic adverse events are relatively infrequent but pooled analyses have shown severe morbidity and fatalities [12, 13]. Myasthenia gravis (MG) is a neuromuscular disease that was reported to have a critical clinical outcome including death post-ICI use in cancer patients. The limited number of cases described in the literature with either new onset MG [14–19], or with a flare of preexisting MG [9], after ICI, limits our ability to characterize the clinical features and outcome of this disease and to optimize its diagnosis and management. Here we describe the clinical and diagnostic features of 65 patients with MG in the setting of ICI, discuss their management strategies, and summarize their clinical outcomes.

## Methods

### Patients

Following institutional review board approval, we searched the institutional databases of the MD Anderson Cancer Center to identify cancer patients who received at least one dose of an FDA-approved ICI (ipilimumab, nivolumab, pembrolizumab, atezolizumab, durvalumab, or avelumab) between January 2011 and December 2018. Patients who had a diagnosis of MG preceding or following the initiation of ICI in the cohort were identified.

We also searched Medline, Web of Science, PubMed ePubs, EMBASE and the Cochrane library through August 2018, with no language or study design restrictions, for case reports, series and observational studies that described patients with cancer and MG receiving ICI. Search strategy and terms are provided in Additional file 1. Titles and abstracts were screened by three independent investigators (in pairs) to identify potentially relevant articles. Then, full text of selected articles was retrieved and reviewed. References of the included articles were hand-searched. A detailed clinical description of each patient was generated. Disagreements were resolved by consensus.

For both MD Anderson and literature identified cases, definite diagnosis of MG was considered on the basis of having ocular and/or systemic muscle weakness, and at least one of the following criteria: (1) elevated titers of anti-acetylcholine receptor (AChR) antibodies, (2) findings suggestive of MG on electrodiagnostic studies, (3) positive edrophonium test, or (4) positive ice pack test. Probable diagnosis of MG was also considered based on the neurologist’s report confirming the diagnosis of MG on the basis of high clinical suspicion alone.

### Methods

For both the MD Anderson and literature identified patients, we extracted data on patient demographics and baseline characteristics (age, gender, type of ICI, type of cancer, and history of comorbidities). We assessed the clinical severity of ICI-related MG using the Myasthenia Gravis Foundation of America (MGFA) classification. Briefly, MGFA class I is defined as weakness isolated to ocular muscles, and MGFA classes II as mild weakness involving any other muscles. MGFA class III and IV are defined by moderate and severe muscle weakness, respectively. MGFA class V is defined as myasthenic crisis involving respiratory failure requiring endotracheal intubation or non-invasive positive pressure mechanical ventilation. Adverse events outcomes were defined as completely resolved, improved, or deteriorated according to the available last follow-up. Tumor response to ICI for MD Anderson patients was categorized using the Response Evaluation Criteria in Solid Tumors 1.1. For patients identified from the literature, response was characterized based on the authors’ report, while remaining cognizant of this limitation. We assessed the quality of the cases identified from the literature using the recommended guidelines for publishing adverse event reports [20]. Data were extracted and quality was assessed by one reviewer and crosschecked by another. Disagreements were resolved by consensus.

### Outcomes analysis

We used descriptive statistics to summarize the data, with median and range for continuous variables and frequencies and percentages for categorical variables. The chi-squared and Fisher’s exact tests were used to compare categorical variables, and the Wilcoxon test or Kruskal-Wallis test was used to compare continuous variables between groups. Timing to respiratory failure after ICI initiation was estimated using the Kaplan-Meier method, and times were right-censored at the last available follow-up.

## Results

A total of 5898 patients received ICI at MD Anderson. Among these, 14 (0.24%) were diagnosed with MG. Of 10,442 unique articles from the literature, 46 publications describing 53 patients met inclusion criteria (Additional file 2: Figure S1), including two patients who had been identified from MD Anderson [21]. Therefore, a total of 65 patients were included in our final analysis; 58 fulfilled the criteria for a definite diagnosis of MG and the remaining patients had probable MG.

### Patient characteristics

Patient demographic and baseline characteristics are shown in Additional file 3: Table S1. The median age was 73 years (range: 34 to 86 years); 42 (65%) were males, and the most common type of cancer was melanoma (48%). Most patients received anti-PD-1 therapy (82%). A preexisting diagnosis of MG was reported in 13 patients (20%). Clinical information for each patient along with the quality appraisal of the cases retrieved from the literature are provided in Additional file 4: Table S2 and Additional file 5: Table S3 respectively.

### ICI-related MG

**Fig. 1:**
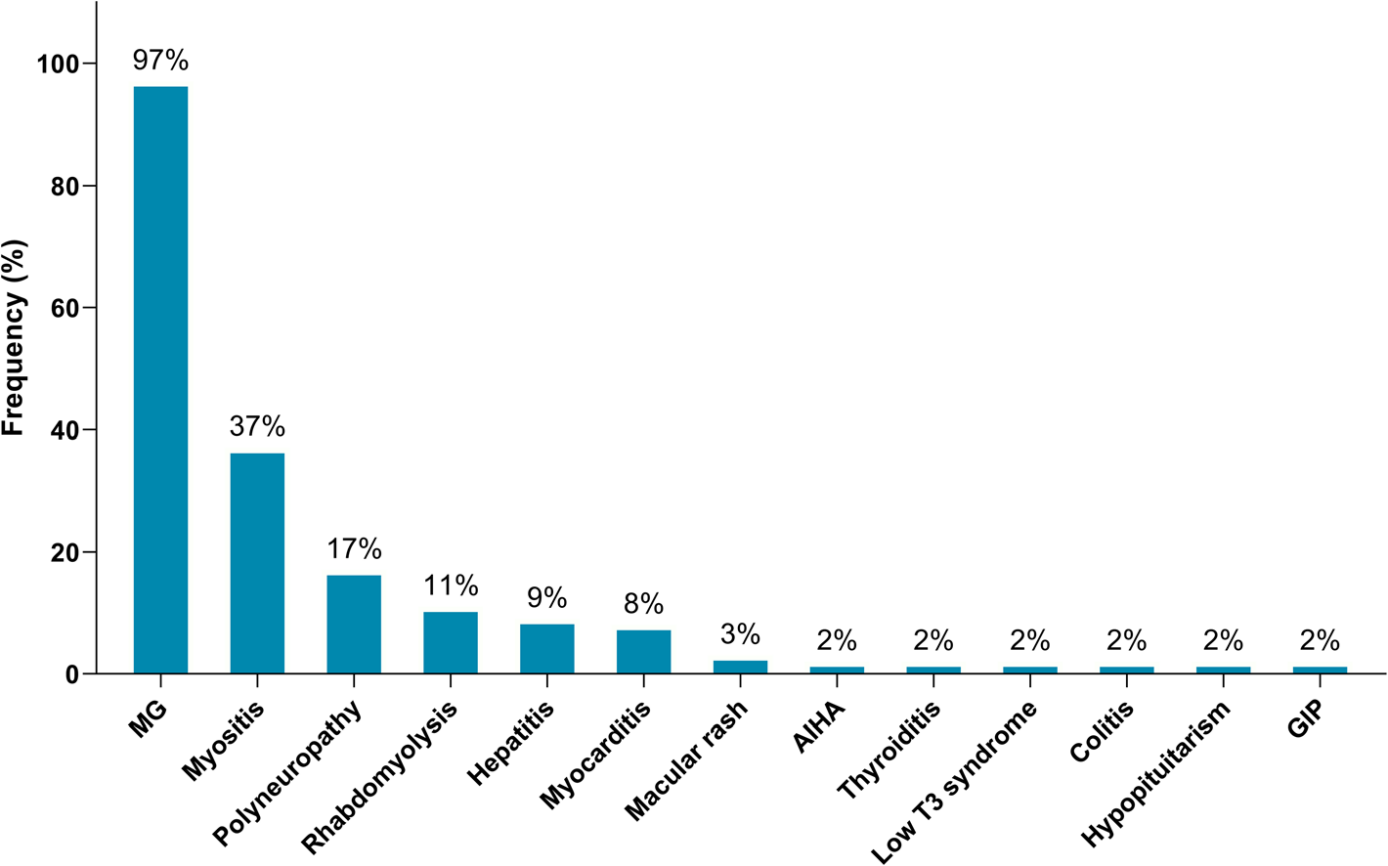
Immune-related adverse events diagnosed in patients following initiation of ICI therapy (*n* = 65). MG = myasthenia gravis; AIHA = autoimmune hemolytic anemia; GIP = granulomatous inflammation of the pleura

**Fig. 2:**
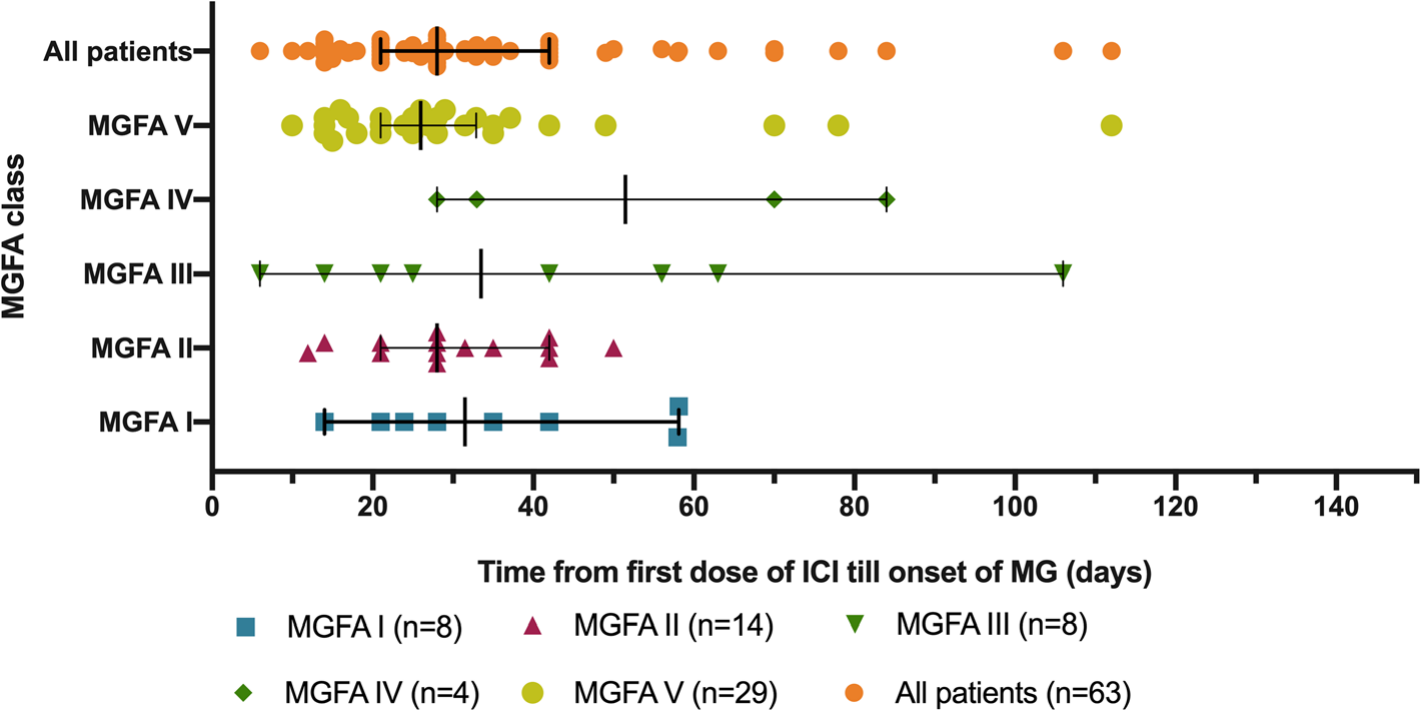
Time from first infusion of immune checkpoint inhibitor to onset of first MG symptom. ICI = immune checkpoint inhibitor; MG = myasthenia gravis; MGFA = Myasthenia Gravis Foundation of America

**Fig. 3:**
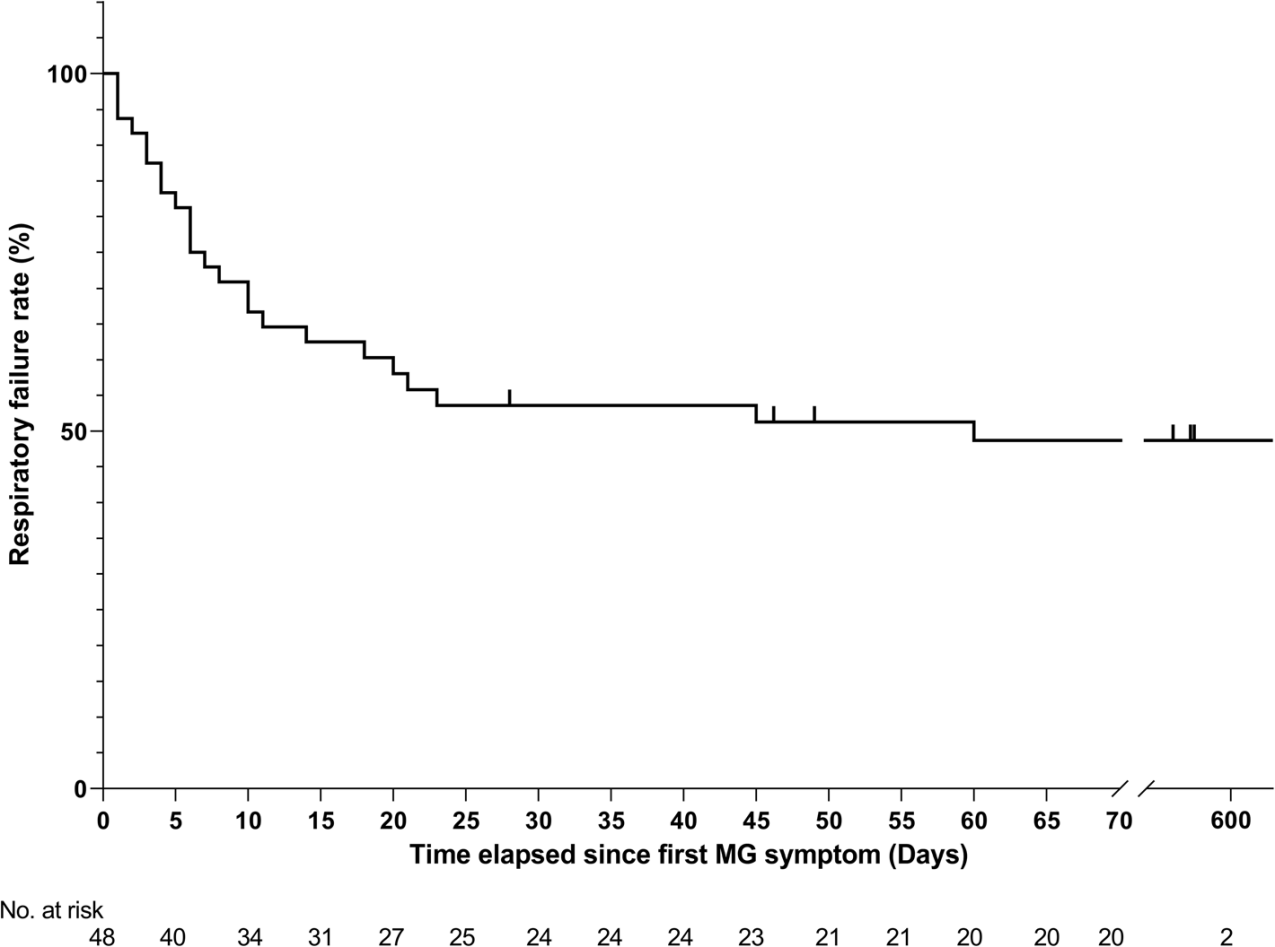
Kaplan-Meier curve for respiratory failure. Of the 63 patients who developed symptoms of myasthenia gravis following initiation of checkpoint inhibitors, the time elapsed since first MG symptom and/or the date of the last follow-up was not available for 15 patients. MG = myasthenia gravis

Of the 65 identified patients, 63 (97%) developed MG symptoms following ICI initiation (52 developed new onset MG and 11 had a flare of their preexisting MG). Overall, 41 (63%) developed moderate to severe muscle weakness (MGFA class III to V) after ICI (Table 1). The most frequent symptoms were ptosis (75%), dyspnea (62%), limb weakness (55%), dysphagia (48%), and diplopia (42%). Concurrent diagnosis of myositis was noted in 24 patients (37%), and myocarditis in five (8%); two had the triad of MG/myositis/myocarditis (Fig. 1). Median time from ICI initiation until the first MG symptom was 4 weeks (range: 6 days - 16 weeks) (Fig. 2). Respiratory failure requiring mechanical ventilation occurred in 29 patients (45%), including 12 who initially presented with severe respiratory compromise, and 17 who progressed to myasthenic crisis after initiation of MG treatment. Patients with MG/myositis/myocarditis seemed to develop respiratory failure more than those with MG only (54% vs. 42%). The median time from first MG symptom till respiratory failure was 7 days (range: 24 h - 60 days) (Fig. 3).

**Table 1:** Clinical characteristics, diagnostic findings, management, and outcomes of ICI-related MG in the whole cohort (*n =* 65) and in the MD Anderson Cancer Center patients (*n =* 14)^a^

Variable	Total Cohort (*n* = 65); n (%)	MDACC (*n* = 14); n (%)
MGFA classification		
I	8 (12)	1 (7)
II	14 (22)	5 (36)
III	8 (12)	0
IV	4 (6)	0
V	29 (45)	7 (50)
Clinical presentation		
Ptosis	49 (75)	11 (79)
Dyspnea	40 (62)	10 (71)
Limb weakness	36 (55)	7 (50)
Dysphagia	31 (48)	7 (50)
Diplopia	27 (42)	4 (29)
Neck weakness	22 (34)	5 (36)
Myalgias	13 (20)	4 (29)
Dysarthria	8 (12)	3 (21)
Facial weakness	8 (12)	3 (21)
Blurry vision	7 (11)	4 (29)
Dysphonia	7 (11)	4 (29)
Generalized weakness	6 (9)	2 (14)
Nasal speech/weakness of the palatal muscles	6 (9)	1 (7)
Incontinence	2 (3)	1 (7)
Diagnostic tools		
Auto antibody panel positive titers		
Anti-AChR	37/56 (66)	5/10 (50)
Anti-Striated muscle	12/18 (67)	6/9 (67)
Muscle enzymes elevation		
CPK	41/49 (84)	9/10 (90)
Troponin	13/14 (93)	6/7 (86)
Edrophonium test positive	4/5 (80)^b^	0
Ice pack test positive	2/4 (50)^b^	0
Electrodiagnostic studies (skeletal muscle EMG, RNS, NCS)		
MG	16/37 (43)^c^	3/9 (33)
Myopathy	6/37 (16)^d^	2/9 (22)
MG and myopathy	6/37 (16)^d^	4/9 (44)
Polyneuropathy	3/37 (8)	0/9
No pathologic findings	6/37 (16)	0/9
Treatment of MG		
Corticosteroids	59/63^e^ (94)	13/13 (100)
Acetylcholinesterase inhibitors	32/63^e^ (51)	7/13 (54)
IVIG	30/63^e^ (48)	9/13 (69)
Plasmapheresis	28/63^e^ (44)	8/13 (62)
Other IST (MMF, rituximab, infliximab or tacrolimus)	10/63^e^ (16)	6/13 (46)
IA	1/63^e^ (2)	1/13 (8)
ICI holding/discontinuation	61/63^e^ (97)	12/13 (92)
MG outcome		
Complete resolution	12/62^e,f^ (19)	6/13 (46)
Improvement	34/62^e,f^ (55)	5/13 (39)
Deterioration	16/62^e,f^ (26)	2/13 (15)
Death	24 (37)	5/14 (36)
MG complications	15 (23)^g^	3/14 (21)
Cancer progression	4 (6)	2/14 (14)
Other comorbidities	3 (5)^h^	0
Unspecified	2 (3)	0

^a^Abbreviations: *MDACC* MD Anderson Cancer Center, *MGFA* Myasthenia Gravis Foundation of America, *Anti-AChR* Anti-Acetylcholine receptor, *CPK* creatine phosphokinase, *EMG* electromyography, *RNS* repetitive nerve stimulation, *NCS* nerve conduction study, *MG* myasthenia gravis, *IVIG* intravenous immunoglobulin, *IST* immunosuppressive therapy, *MMF* mycophenolic acid, *IA* immunoadsorption, *ICI* immune checkpoint inhibitor. Numbers are rounded to the nearest whole number

^b^One patient had a partially positive test result

^c^Three patients also had findings suggestive of polyneuropathy

^d^Two patients also had findings suggestive of polyneuropathy

^e^Two patients with pre-existing MG did not develop a flare of their disease after ICI initiation and were excluded from the analysis

^f^Data were not reported for one patient

^g^Twelve patients died from respiratory failure, one patient died from hospital acquired pneumonia following hospitalization and two others died from worsening general status from severe dysphagia

^h^One patient died following acute hypercapnic respiratory failure unrelated to MG according to the authors, one patient died from complications of a preexisting heart disease and one patient died from aspiration pneumonia 1 month after discharge

#### Diagnostic features

Elevation of anti-AChR antibodies was reported in 37/56 tested patients (66%) (median: 1.64 nmol/L, range: 0.05–98 nmol/L) (Table 1). Of note, three patients were found to have positive AChR antibody in retrospective blood samples that were drawn before ICI initiation, but their antibody titers increased at least 2-fold after ICI [22–24]. Anti-striated antibodies were detected in 12/18 tested patients (67%), and 41/49 tested patients (84%) had elevated creatine phosphokinase (CPK) (median 2638 IU/L, range: 418 to 19,794 IU/L). Those with elevated CPK seemed to develop respiratory failure more than those with normal levels (56% vs. 38%). Details on other diagnostic tools are summarized in Additional file 6: Table S4.

Electrodiagnostic studies were performed in 37 patients and detected features of MG in 15 (41%), and both MG and myopathy in six (16%). Computed tomography was negative for thymoma, and magnetic resonance imaging excluded brain metastasis or acute intracranial events. Transthoracic echocardiography showed left ventricular dysfunction in four patients (27%) who had an overlapping diagnosis of myositis/myocarditis, while electrocardiography showed diffuse ST elevation, premature ventricular contractions, right bundle branch block, or ventricular tachycardia in five others (34%).

Skeletal muscle biopsy was performed in seven patients and showed inflammatory infiltrates in five (71%). Three others had myocardial biopsy, which revealed inflammatory infiltrates in all. The inflammatory infiltrates in both skeletal and myocardial biopsies consisted of CD8+ and CD4+ T lymphocytes as well as B lymphocytes and macrophages.

#### Management and outcomes

Of the 63 patients who developed ICI-related MG, 96% required hospitalization. Overall, corticosteroids (3–1000 mg/day) were used in 59 patients (94%) (Table 1). Acetylcholinesterase inhibitors were used in 32 (51%), intravenous immunoglobulin (IVIG) in 30 (48%), plasmapheresis (PLEX) in 28 (44%), and other immunosuppressants in 10 (16%). Additionally, invasive ventilation was used in 12 patients (19%) and non-invasive positive pressure ventilation in 14 (22%); three others refused intubation and opted for palliative care. Only 10 patients were successfully weaned from mechanical ventilation including three who still required oxygen therapy. One patient with myocarditis also required temporary implantation of a pacemaker [25], and another one required intervention with an intra-aortic balloon pump [26]. Discontinuation or withholding of ICI was recommended in 61 patients (97%), the remaining two continued ICI after resolution of symptoms with steroids [27].

**Fig. 4:**
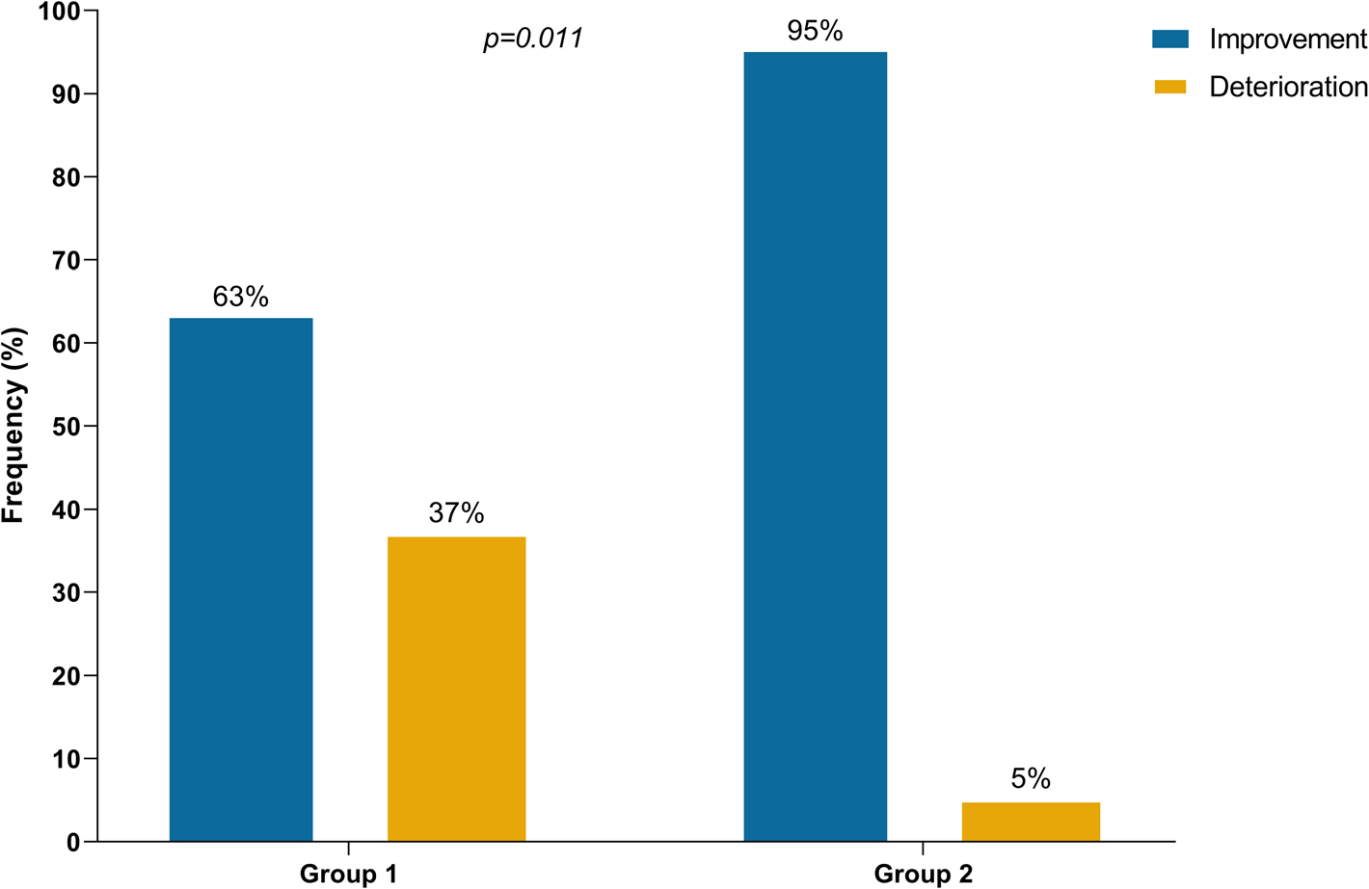
Outcomes of immune checkpoint inhibitor-related myasthenia gravis according to first-line treatment. Group 1: Patients who received steroids without concurrent intravenous immunoglobulin or plasmapheresis in first-line treatment (n=38). Group 2: Patients who received intravenous immunoglobulin or plasmapheresis regardless of steroids in first-line treatment (n=19)

Overall, MG symptoms completely resolved in 12 patients (19%), improved in 34 (55%), and worsened in 16 (26%) (Table 1). Information on both the sequence of treatments for MG and outcome at last follow-up were available for 59 patients. Of 38 patients who received steroids only as first line therapy, 24 (63%) had improvement of symptoms. In the remaining 14 patients who progressed to respiratory failure, IVIG or PLEX was added as a second line therapy for 12 patients but with no improvement. Of note, four of these 14 patients had initially presented with ocular symptoms but eventually progressed to myasthenic crisis after initiation of steroid (ranging from 30 mg to 1000 mg per day). In contrast, of 19 patients who received IVIG or PLEX (regardless of steroid) as first line, 18 (95%) had improvement of symptoms (*p = 0.011*) (Fig. 4). Of note, the upfront use of IVIG or PLEX in those patients may have been triggered by early development of severe respiratory/bulbar symptoms in 17 patients, and may have been based on the prescriber’s preference in two others who presented with only mild symptoms. Additionally, one patient with ocular symptoms was treated by holding ICI, and another one with mild weakness was treated by acetylcholinesterase inhibitor leading to improvement. Data on maintenance therapy for MG after discharge were available for 31 patients. Of those, 26 (84%) were on steroid taper protocols, 10 (32%) were receiving acetylcholinesterase inhibitors, five (16%) IVIG, one mycophenolic acid, and another one rituximab. Death was reported in 24 patients (37%), primarily because of MG complications in 15 patients (23%) after a median of 6 weeks (range: 3–26.5 weeks) of the initial MG symptoms. Of the 15 patients who died because of MG complications, two had MG alone, and 13 had elevated CPK levels including nine who were diagnosed with MG overlapping with myositis/myocarditis. Overall, patients who were tested for CPK and/or troponin seemed to have a higher MG deterioration rate than those who were not tested (29% vs. 13%), and a higher mortality rate primarily because of MG complications (29% vs. 6%) (Additional file 7: Table S5).

Information on the tumor response to ICI was available for 20 patients with melanoma. Ten (50%) achieved partial or complete response, five (25%) had stable disease, while five others (25%) had tumor progression.

Notably, the clinical presentation, diagnostic findings, management, and clinical outcomes of ICI-related MG did not differ when we excluded the patients who were identified from the literature (Table 1) nor the patients diagnosed with probable MG from our cohort (Additional file 8: Table S6).

#### Rechallenge or continuation of ICI treatment

Re-administration of ICI was reported for six patients after resolution of MG symptoms. In three the initial MG symptoms were limited to ocular symptoms or mild weakness, while the other three had more severe weakness. After symptom resolution, all patients were maintained on prednisone, pyridostigmine and/or IVIG at ICI re-administration. Time from the first MG symptom until ICI re-administration ranged from 7 days to 17.75 months. Five patients were treated with the same initial agent (anti-PD-1) and one switched from ipilimumab to pembrolizumab. Upon ICI re-administration, none of the patients had recurrence of symptoms. Two of these patients eventually had partial or complete tumor response, one had stable disease, and three had progressive disease.

#### Patients with preexisting MG

Thirteen patients had pre-existing MG; eight (67%) were treated with immunosuppressants (steroids, mycophenolate mofetil, azathioprine), IVIG, and/or acetylcholinesterase inhibitor before ICI initiation. The time interval between diagnosis of MG and ICI initiation was 5.3 years (1–20 years). At ICI initiation, modification of the baseline treatment (reducing immunosuppression dose and/or acetylcholinesterase inhibitors or adding IVIG) was recommended for four patients, and only one had active preexisting MG symptoms. Of the 13 patients, 11 (85%) had disease flare after ICI initiation. One patient (8%) developed ocular symptoms (MGFA classes I) and ten others (76%) developed more severe weakness (MGFA III, IV and V) (Table 2). MG flare was fatal in two patients including one who was receiving maintenance therapy at ICI initiation. There were no significant differences in median times from ICI initiation to onset of MG symptoms, MGFA class, clinical manifestations, diagnostic findings, management, nor clinical outcomes between patients with preexisting MG and those with new onset disease that manifested clinically only after ICI initiation (Table 2).

**Table 2:** Clinical characteristics, diagnostic findings, management, and outcomes of ICI-related MG in patients with pre-existing MG (*n =* 13) and those with new onset disease (*n =* 52)^a^

Variable	New Onset (*n =* 52); *n* (%)	Pre-existing MG (*n =* 13); *n* (%)	*p* value
Median time from ICI initiation to onset of MG symptoms (weeks)			0.27
	4	4.7	
MGFA classification			0.161
I	7 (14)	1 (8)	
II	14 (27)	0	
III	6 (12)	2 (15)	
IV	2 (4)	2 (15)	
V	23 (44)	6 (46)	
Clinical presentation			
Ptosis	43 (83)	6 (46)	0.01
Dyspnea	32 (62)	8 (62)	1
Limb weakness	30 (58)	6 (46)	0.54
Dysphagia	24 (46)	7 (54)	0.76
Diplopia	22 (42)	5 (39)	0.8
Neck weakness	20 (39)	2 (15)	0.19
Myalgias	13 (25)	0	0.06
Blurry vision	7 (14)	0	0.33
Dysarthria	6 (12)	2 (15)	0.65
Generalized weakness	6 (12)	0	0.34
Dysphonia	6 (12)	1 (8)	1
Facial weakness	5 (10)	3 (23)	0.19
Nasal speech/weakness of the palatal muscles	5 (10)	1 (8)	1
Incontinence	2 (4)	0	1
Diagnostic tools			
Auto antibody panel positive titers			
Anti-AChR	32/50 (64)	5/6 (83)	0.65
Anti-Striated muscle	12/18 (67)	0/0	N/A
Muscle enzymes elevation			
CPK	37/44 (84)	4/5 (80)	1
Troponin	12/13 (92)	1/1 (100)	1
Edrophonium test positive	5/5 (100)	0	N/A
Ice pack test positive	2/4 (50)	0	N/A
Electrodiagnostic studies (skeletal muscle EMG, RNS, NCS)			
MG	14/34^b^ (41)	2/3 (67)	0.56
Myopathy	6/34^c^ (18)	0/3	0.58
MG and myopathy	6/34^c^ (18)	0/3	0.33
Polyneuropathy	2/34 (6)	1/3 (33)	0.23
No pathologic findings	6/34 (18)	0/3	0.58
Treatment of MG			
Corticosteroids	48 (92)	11/11^d^ (100)	1
Acetylcholinesterase inhibitors	25 (48)	7/11^d^ (64)	0.35
IVIG	25 (48)	5/11^d^ (46)	0.87
Plasmapheresis	22 (42)	6/11^d^ (55)	0.52
Other IST (MMF, rituximab, infliximab or tacrolimus)	7 (13)	3/11^d^ (27)	0.36
IA	3 (6)	0/11^d^	1
ICI holding/discontinuation	51 (98)	10/11^d^ (91)	0.32
MG outcome			
Complete resolution	8/51 (16)	4/11^d^ (36)^e^	0.2
Improvement	30/51^e^ (59)	4/11^d^ (36)	0.2
Deterioration	13/51^e^ (26)	3/11^d^ (27)	1
Death	21 (40)	3 (23)	0.34
MG complications	13 (25)	2 (15)	0.72
Cancer progression	4 (8)	0	0.58
Other comorbidities	2 (4)	1 (8)	0.5
Unspecified	2 (4)	0	1

^a^Abbreviations: *MG* myasthenia gravis, *ICI* immune checkpoint inhibitor, *MGFA* Myasthenia Gravis Foundation of America, *Anti-AChR* Anti-Acetylcholine receptor, *CPK* creatine phosphokinase, *EMG* electromyography, *RNS* repetitive nerve stimulation, *NCS* nerve conduction study, IVIG intravenous immunoglobulin, *IST* immunosuppressive therapy, *MMF* mycophenolic acid, *IA* immunoadsorption. Numbers are rounded to the nearest whole number

^b^Three patients also had findings suggestive of polyneuropathy

^c^Two patients also had findings suggestive of polyneuropathy

^d^Two patients with pre-existing MG did not develop a flare of their disease after ICI initiation and were excluded from the analysis

^e^Data were not reported for one patient

Only two patients with preexisting MG did not show any signs of disease exacerbation after ICI initiation. Both had no active MG symptoms at ICI initiation and were maintained on prednisone 10 mg or pyridostigmine 120 mg. Of 5 melanoma patients, 3 (80%) achieved partial response.

#### Patients with ICI-related MG compared to idiopathic MG (iMG)

Patient demographics, MGFA classification, time to class IV/V, rate of MG/myositis/myocarditis overlap, and type of autoantibodies observed in patients with ICI-related MG compared to iMG are shown in Table 3. MGFA class IV/V MG occurred in more than half of our patients (51%) which is much higher than what has been recently reported in patients with iMG (2–10%) [26, 28]. The median time from first MG symptom to class IV/V was 7 days (range: 24 h to 60 days) in our cohort, while in those with iMG, progression to class IV/V typically occurs within 2–3 years [29, 30]. The overlap with myositis/myocarditis was also much higher compared to patients with iMG (42% vs. 0.9%) [31]. Additionally, positive anti-striated muscle antibodies were more frequently observed in our patients compared to non-thymoma patients with iMG [32].

**Table 3:** Demographics and clinical characteristics of patients with ICI-related MG and those with idiopathic MG^a^

Variable	ICI-related MG	iMG [26, 28–30, 32, 50]
Mean age (years)	71 ± 9.7	60.0 ± 18.8
Female gender (%)	35%	59%
MGFA classification		
I	12%	52%
II	22%	43%
III	12%	3%
IV	6%	2%
V	45%	0%
Time from first symptom to Class IV/V	1–60 days (Median: 7 days)	2–3 years
Overlap with myositis/myocarditis	42%	0.9%
Antibodies		
Anti-AChR	66%	83.2%
Anti-striated muscle	67%	36%^b^
Anti-MuSK	3%	2.3%

^a^Abbreviations: *ICI* immune checkpoint inhibitors, *MG* myasthenia gravis, *iMG*, idiopathic myasthenia gravis, *MGFA* myasthenia gravis foundation of America, Anti-AChR Anti-Acetylcholine receptor, *Anti-MuSK* Anti-muscle specific kinase. Numbers are rounded to the nearest whole number

^b^For patients with non-thymomatous MG

## Discussion

Our data support that MG is a life-threatening irAE with an acute onset and rapid deterioration shortly after ICI initiation. Approximately two-thirds of our patients developed severe muscle weakness (MGFA class V) with respiratory dysfunction requiring mechanical support in 45%.

Our findings support that ICI-related MG has several unique features compared to iMG, which typically manifests as a milder disease where most patients fall under MGFA classes I and II [26], and which has a slower clinical deterioration course that may take 2–3 years to progress to class V [29, 30]. Age at diagnosis of ICI-related MG in our cohort was also significantly older than in patients with iMG [26]. This raises the question of whether elderly patients with cancer are more susceptible to this particular adverse event. We also observed that more than one-third of our patients developed MG overlapping with myositis/myocarditis; those patients seemed to have more severe symptoms and worse clinical outcomes than patients with MG alone. Of note, we believe that myositis may have been underdiagnosed in our cohort, as many patients reported in the literature had myalgias and elevated CPK but the concurrent diagnosis of myositis was not discussed. The overlap of these two entities, in addition to myocarditis, has been described in only 0.9% of patients with iMG, and is frequently associated with thymoma [31, 33, 34], progression to myasthenic crisis [31, 35], and positive striational antibodies, the latter are believed to be markers of poor prognosis [36].

The underlying immunobiology is not well studied in ICI-related MG. Gene expression analysis of peripheral blood mononuclear cells was performed before and after nivolumab in one patient who developed MG/myositis/myocarditis, and revealed an increased expression of CD8 and cytolytic activity markers, whereas CD4+ T-cell and T regulatory cell activity seemed suppressed [22]. In another patient with nivolumab-related MG/myositis, peripheral blood lymphocyte analysis showed an elevated CD8:CD4 ratio of 1.4 [37], these findings can possibly be related to the activity of nivolumab regardless of induction of MG.

For almost all patients in our cohort (96%), hospitalization and corticosteroids were recommended, and severity of symptoms at initial presentation failed to predict the course of disease. For instance, few patients initially presented with mild ocular symptoms but suddenly progressed to respiratory failure despite initiation of steroids. Our data suggest that patients who received IVIG or PLEX as a first-line treatment experienced better MG outcomes than those who received steroids alone (95% vs 63% improvement of MG symptoms, *p = 0.011)*. Our results also suggest that IVIG or PLEX may be most effective when used as the first-line regimen, as several patients who deteriorated after initial use of steroids failed to improve despite a second-line use of IVIG or PLEX. In contrast to our findings, the clinical practice guidelines for management of ICI-related MG recommend adding IVIG or PLEX if patients had no improvement/worsening on steroid alone, or presented with severe symptoms (MGFA class III to V) [38, 39]. Given the acute onset and rapid deterioration of ICI-related MG, we recommend the early use of IVIG or PLEX in the first-line regimen regardless of the severity of initial symptoms. In fact, the use of steroids as a sole first-line therapy might not be ideal for management considering that these drugs might take several weeks to show clinical response [40]. Additionally, it is well known that steroid use can cause an acute exacerbation of iMG symptoms [41]. Although this worsening has been described as transient, it occurs in 50% of patients, and includes a serious risk of progression to respiratory failure [42]. On the other hand, the use of IVIG and PLEX have led to favorable outcomes in most patients with severe iMG [43–45], and their early use is recommended preceding or simultaneously with steroids to overcome the risk of a transient worsening, especially in patients with severe disease [40, 46, 47]. One should keep in mind that the use of steroids alone in ICI-related MG might be associated with an even worse prognosis as these patients might not be able to survive a transient worsening of symptoms following steroids because of their old age and advanced malignancy. Of note, these patients typically require steroids at higher doses compared to iMG because of the concurrent myositis/myocarditis as well as other organ toxicities. Moreover, the role of steroids in controlling immune dysregulation in these patients might be limited by the constant presence of the original trigger; the circulating ICIs, as their half-life ranges from 14.7 to 27.3 days depending on the agent [48, 49]. Therefore, elimination of the pathogenic antibodies and ICI mAbs from the sera of patients using IVIG or PLEX could mediate a faster improvement of symptoms. Given the small number of patients and the retrospective nature of our study, we were unable to estimate the frequency of a steroid induced exacerbation in our cohort. However, our data might suggest the need to reassess the current recommendations for management of ICI-related MG.

Death primarily due to MG complications was reported in 23% of our cohort. In regards to tumor response, our data show that the clinical benefit rate in melanoma patients reaches up to 75% suggesting a possibly enhanced antitumor immune response. Our findings could also suggest that short-term immune modulation using IVIG or PLEX along with steroids might not alter the durability of tumor response to ICI.

The decision to rechallenge patients with ICI-related MG using an ICI is a dilemma that we do not currently have an answer for. Although our data partially suggest a safe re-administration of ICI after resolution of MG symptoms and while on maintenance therapy, the number of patients was too small to infer with confidence any definitive conclusions. It is well-known that iMG is mostly characterized to have a “monophasic” clinical course. However, whether ICI-related MG have similar clinical pattern remains unclear, since our data have shown that all patients who were rechallenged or continued on ICI were kept on immunosuppressive therapy.

We did not observe differences in the clinical course and outcomes of ICI-related MG between patients who experienced flare of a preexisting MG and those with a new onset disease. Given the lack of prospective cohort studies, the true incidence rate of MG flare after ICI in patients with a prior diagnosis of MG cannot be currently estimated. Moreover, whether patients who develop new onset MG, in fact, have had a subclinical autoimmunity that manifested only after exposure to ICI remains questionable. Therefore, further longitudinal studies are needed to validate these findings and judiciously evaluate if screening for MG should be considered before initiation of ICI.

To our knowledge, our study represents the largest cohort of patients with ICI-related MG, and the most comprehensive systematic review of the literature. Although it is limited by its retrospective nature, our findings help clinicians gain familiarity with the severity and the rapidly progressive course of ICI-related MG, and suggest a possibly enhanced management recommendation with early use of IVIG/PLEX. Additionally, our data provide a safety signal that would help clinicians consider the risks and benefits for each individual patient predominantly elderly and those receiving ICI as an adjuvant therapy.

## Conclusions

In summary, MG in the setting of ICI use is an acute and life-threatening adverse event with varied clinical presentations and rapid deterioration. Therefore, it is critical that patients, primarily those receiving adjuvant ICI, be aware of this possible complication. The healthcare providers should also be mindful of the need for a multidisciplinary approach and a multimodal aggressive therapy. Preclinical studies are warranted to enhance our understanding of the immunobiology of this irAE, so we can carefully evaluate the risk benefit ratio of ICI use in susceptible patients. Multi-institutional clinical trials are needed to establish the ideal therapeutic approach for this life-threatening complication.

## Supplementary information

**Additional file 1.** Search Strategy.

**Additional file 2: Figure S1.** Study Selection Flowchart.

**Additional file 3: Table S1.** Patient Demographics and baseline Characteristics.

**Additional file 4: Table S2**. Clinical, diagnostic and outcome features of patients from MD Anderson and patients from the literature.

**Additional file 5: Table S3.** Quality Appraisal of the Literature Reported Cases.

**Additional file 6: Table S4.** Diagnostic tools used in patients with suspected ICI-related MG.

**Additional file 7: Table S5**. MG outcomes and survival data for patients who were tested for muscle enzymes elevation and those who were not.

**Additional file 8: Table S6.** Clinical characteristics, diagnostic findings, management, and outcomes of ICI-related MG in the whole cohort and in patients with a definite diagnosis of MG.

## Data Availability

The datasets supporting the conclusions of this article are included within the article and its additional files.

## Abbreviations

AChR: Acetylcholine receptor
CPK: Creatine phosphokinase
CTLA-4: T-cell lymphocyte associated antigen-4
ICI: Immune checkpoint inhibitor
iMG: Idiopathic myasthenia gravis
irAE: Immune related adverse event
IVIG: Intravenous immunoglobulin
MG: Myasthenia gravis
MGFA: Myasthenia gravis foundation of America
PD-1: Programmed cell death-1
PD-L1: Programmed cell death-ligand 1
PLEX: Plasmapheresis
