# Supplementary material for: Immune checkpoint inhibitor related myasthenia gravis: single center experience and systematic review of the literature
Source: J Immunother Cancer. 2019 Nov 21;7:319. doi: 10.1186/s40425-019-0774-y (PMC6868691; doi:10.1186/s40425-019-0774-y)
Supplement: Supplementary file 2 — Additional file 2: Figure S1. Study Selection Flowchart. [file 40425_2019_774_MOESM2_ESM.docx]

**Figure S1.** Study Selection Flowchart

**Articles identified through bibliography hand-searching (n =**15**)**

**Articles identified through databases (n = 21,233)**

- Medline (n = 2,320)
- EMBASE (n = 9,556)
- Web of Science (n = 3,571)
- Cochrane CENTRAL (n = 3,407)
- PubMed ePubs (n =2,379)

**Unique articles after duplicates (10,806) were removed (n = 10,442)**

**Articles excluded (n =8,225)**

- Clinical trials (n=2,338)
- Basic science (n =581)
- Not the drug of interest (n = 148)
- Not the population of interest (n = 71)
- Observational studies/trials/case series not reporting individual case descriptions (n = 2,603)
- Two or more exclusion criteria (n = 2,482)
- Irretrievable (n = 2)

**Full-text articles assessed for eligibility**

**(n =2,217)**

**Full-text articles excluded (n =2,171)**

- No diagnosis of myasthenia gravis

**Publications included (n=46)**

**Reported cases (n=53)**
