# Supplementary material for: Immune checkpoint inhibitor related myasthenia gravis: single center experience and systematic review of the literature
Source: J Immunother Cancer. 2019 Nov 21;7:319. doi: 10.1186/s40425-019-0774-y (PMC6868691; doi:10.1186/s40425-019-0774-y)
Supplement: Supplementary file 3 — Additional file 3: Table S1. Patient Demographics and baseline Characteristics. [file 40425_2019_774_MOESM3_ESM.docx]

**Table S1.** Patient Demographics and baseline Characteristics (*n=* 65).^a^

| **Patient characteristic** | **No. (%)** |
| --- | --- |
| Median age, years (range) | 73 (34–86) |
| Sex |  |
| Male | 42 (65) |
| Female | 23 (35) |
| Current cancer |  |
| Metastatic melanoma | 31 (48) |
| Non-small cell lung cancer | 15 (23) |
| Renal cell carcinoma | 7 (11) |
| CRC | 1 (2) |
| Mesothelioma | 1 (2) |
| Neuroendocrine carcinoma of the trachea | 1 (2) |
| Prostate adenocarcinoma | 1 (2) |
| Sarcoma | 1 (2) |
| SCC of the head and neck | 1 (2) |
| SCC of the thymus | 1 (2) |
| SCLC | 1 (2) |
| Thymoma | 1 (2) |
| Urachal cancer | 1 (2) |
| Urothelial carcinoma of the bladder | 1 (2) |
| Uterine carcinosarcoma | 1 (2) |
| Checkpoint inhibitor used |  |
| Anti-PD-1 |  |
| Nivolumab^b^ | 33 (51) |
| Pembrolizumab^c^ | 20 (31) |
| Anti-CTLA4 |  |
| Ipilimumab | 5 (8) |
| Combination of ipilimumab and nivolumab | 4 (6) |
| Anti-PDL1 |  |
| Atezolizumab | 2 (3) |
| Durvalumab^d^ | 1 (2) |
| Preexisting MG | 13 (20) |

^a^Abbreviations: CRC, colorectal cancer; SCC, squamous cell carcinoma; SCLC, small cell lung cancer; PD1, programmed cell death protein 1; CTLA4, cytotoxic T lymphocyte associated protein 4; PDL1, programmed cell death ligand 1; MG, myasthenia gravis. Numbers are rounded to the nearest whole number.

^b^One patient received sitravatinib and another received NKTR-214 in combination with nivolumab.

^c^One patient received azacitadine in combination with pembrolizumab.

^d^The patient received tremelimumab in combination with durvalumab.
