# Supplementary material for: Immune checkpoint inhibitor related myasthenia gravis: single center experience and systematic review of the literature
Source: J Immunother Cancer. 2019 Nov 21;7:319. doi: 10.1186/s40425-019-0774-y (PMC6868691; doi:10.1186/s40425-019-0774-y)
Supplement: Supplementary file 4 — Additional file 4: Table S2. Clinical, diagnostic and outcome features of patients from MD Anderson and patients from the literature. [file 40425_2019_774_MOESM4_ESM.docx]

**Table S2**. Clinical, diagnostic and outcome features of patients from MD Anderson and patients from the literature.

| Patient ID/Age/Gender | Type of cancer | Preexisting MG | Type of ICI | Time to onset (weeks) | Clinical manifestations (MGFA class) | Anti-AchR/striational antibodies | CPK (value in IU/L) | Myositis/Myocarditis | Treatment | ICI-related MG outcome | Tumor response to ICI |
| --- | --- | --- | --- | --- | --- | --- | --- | --- | --- | --- | --- |
| 1/61/M | Melanoma | Yes | Nivolumab | N/A | No exacerbation | N/A | N/A | -/- | N/A | N/A | NR |
| 2/85/M | Melanoma | Yes | Pembrolizumab | N/A | No exacerbation | N/A | N/A | -/- | N/A | N/A | NR |
| 3/74/F | Melanoma | No | Nivolumab | 4 | Ptosis, diplopia (MGFA I) | +/NR | + (654) | -/- | GC, pyridostigmine | Improvement | SD |
| 4/53/M | Melanoma | Yes | Nivolumab | 6 | Ptosis, diplopia (MGFA I) | ND/ND | ND | -/- | GC, pyridostigmine | Complete resolution | PR |
| 5/81/M | Melanoma | No | Pembrolizumab | 8.28 | Ptosis (MGFA I) | -/NR | ND | -/- | GC | Improvement | PR |
| 6/85/F | Melanoma | No | Pembrolizumab | 3.00 | Ptosis, diplopia (MGFA I) | -/NR | - (NR) | -/- | GC, pyridostigmine, IVIG | Complete resolution | NR |
| 7/68/M | Melanoma | No | Pembrolizumab | 5.00 | Ptosis, diplopia (MGFA I) | -/NR | ND | -/- | GC | Improvement | CR |
| 8/65/M | NSCLC | No | Nivolumab | 3.42 | Ptosis, diplopia, blurry vision, (MGFA I) | -/NR | NR | -/- | Pyridostigmine | Complete resolution | NR |
| 9/57/M | NSCLC | No | Nivolumab | 8.3 | Ptosis (MGFA I) | +/NR | - (NR) | -/- | GC, pyridostigmine | Improvement | NR |
| 10/73/M | Renal cancer | No | Nivolumab | 2.00 | Ptosis, Myalgia, (MGFA I) | NR/NR | + (NR) | -/+ | GC | NR | NR |
| 11/70/F | Melanoma | No | Ipilimumab | 4 | Ptosis, dysphagia, limb/neck weakness, myalgia, (MGFA II) | +/+ | + (1200) | +/- | GC, PLEX | Improvement | SD |
| 12/73/F | Melanoma | No | Ipilimumab | 3.00 | Limb weakness, dyspnea (MGFA II) | +/NR | ND | -/- | GC, pyridostigmine | Improvement | PD |
| 13/72/F | Melanoma | No | Ipilimumab | 7.14 | Ptosis, blurry vision, dysphagia, myalgia, dyspnea (MGFA II) | +/+ | + (1389) | +/- | GC, PLEX | Complete resolution | PD |
| 14/60/M | Melanoma | No | Ipilimumab/Nivolumab | 2 | Ptosis, diplopia, blurry vision, dysphagia, limb/neck weakness, myalgia, generalized weakness, dyspnea (MGFA II) | -/+ | + (10644) | +/- | GC, pyridostigmine, PLEX, IVIG | Improvement | SD |
| 15/75/F | Melanoma | No | Nivolumab | 4.5 | Limb weakness (MGFA II) | +/NR | + (1773) | -/- | GC | Improvement | NR |
| 16/72/M | Melanoma | No | Nivolumab | 4 | Ptosis, dysphagia, dysarthria, dyspnea (MGFA II) | ND/ND | ND | -/- | GC | Complete resolution | PD |
| 17/67/M | Melanoma | No | Nivolumab | 6 | Ptosis, diplopia, dysphagia, dysphonia, ;imb/neck weakness (MGFA II) | -/- | - (NR) | -/- | GC, IVIG | Improvement | Not assessed |
| 18/86/F | Melanoma | No | Pembrolizumab | 6.00 | Ptosis, dysphagia, (MGFA II) | -/NR | ND | -/- | GC | Improvement | CR |
| 19/57/M | NSCLC | No | Ipilimumab/Nivolumab | 6.00 | Ptosis, dysphagia, dysphonia, limb/neck weakness, dyspnea (MGFA II) | +/NR | + (2682) | +/- | GC, pyridostigmine | Death from MG complications^b^ | NR |
| 20/81/M | NSCLC | No | Nivolumab | 4.00 | Ptosis, nasal speech/weakness of the palatal muscles, limb weakness (MGFA II) | +/NR | - (NR) | -/- | GC | Complete resolution | PD |
| 21/83/M | NSCLC | No | Pembrolizumab | 4 | Ptosis, diplopia, neck weakness, myalgia, (MGFA II) | -/- | + (4361) | +/- | GC, pyridostigmine | Complete resolution | CR |
| 22/69/M | Prostate adenocarcinoma | No | Ipilimumab/Nivolumab | 5 | Ptosis, incontinence (MGFA II) | -/+ | + (1402) | +/- | GC, PLEX, IVIG, MMF, rituximab | Complete resolution | SD |
| 23/79/F | Renal cancer | No | Nivolumab | 2.00 | Ptosis, limb weakness (MGFA II) | -/NR | + (5350) | +/- | ICI hold | Improvement | NR |
| 24/75/M | SCC of the head and neck | No | Nivolumab | 3 | Ptosis, limb weakness (MGFA II) | +/+ | + (2593) | +/- | GC, pyridostigmine, PLEX | Deterioration | PD |
| 25/69/F | Melanoma | No | Ipilimumab | 3 | Ptosis, diplopia, blurry vision, dysphagia, neck weakness, dyspnea (MGFA III) | +/NR | ND | -/- | GC, pyridostigmine, PLEX | Improvement | PR |
| 26/74/M | Melanoma | No | Ipilimumab | 6.00 | Diplopia, limb weakness, dyspnea (MGFA III) | -/NR | NR | -/- | GC, pyridostigmine | Improvement | NR |
| 27/79/M | Melanoma | Yes (Ocular) | Nivolumab | 15.14 | Diplopia, dysphagia, facial weakness (MGFA III) | +/NR | + (1627) | -/- | GC | Complete resolution | PR |
| 28/62/F | Neuroendocrine carcinoma of the trachea | Yes (Ocular) | Nivolumab | 3.57 | Ptosis, diplopia, dysphagia, dysarthria, limb/facial weakness (MGFA III) | +/NR | + (14229) | +/- | GC | Improvement | PR |
| 29/79/M | NSCLC | No | Nivolumab | 2.00 | Ptosis, dysphagia, limb wekaness (MGFA III) | +/+ | + (2431) | +/- | GC, ambemonium | Improvement | PR |
| 30/75/M | NSCLC | No | Nivolumab | 0.85 | Dysphagia, nasal speech/weakness of the palatal muscles, limb weakness (MGFA III) | -/NR | - (NR) | -/- | IVIG | Improvement | NR |
| 31/34/F | SCC of the thymus | No | Pembrolizumab | 8 | Ptosis, diplopia, dysphagia, dysphonia, nasal speech/weakness of palatal muscles, facial/neck weakness dyspnea (MGFA III) | +/- | + (2125) | +/- | GC, IVIG, PLEX | Improvement | SD |
| 32/71/F | Uterine carcinocarcinoma | No | Pembrolizumab | 9 | Ptosis, diplopia, dysphagia, dysarthria, limb/neck weakness (MGFA III) | -/NR | + (1200) | -/- | GC, pyridostigmine | Improvement | PD |
| 33/59/F | Melanoma | Yes | Pembrolizumab | 12 | Dysphagia, dysphonia, limb weakness, dyspnea (MGFA IV) | -/NR | NR | -/- | GC, IVIG, PLEX, PEG | Improvement | NR |
| 34/69/F | Melanoma | No | Pembrolizumab | 10.00 | Ptosis, generalized weakness, dyspnea (MGFA IV) | -/NR | + (NR) | -/- | GC, pyridostigmine, PLEX | Death from MG complications | PR |
| 35/74/M | Melanoma | Yes | Pembrolizumab | 4.71 | Ptosis, dysphagia, limb weakness, dyspnea (MGFA IV) | ND/NR | ND | -/- | GC, pyridostigmine, IVIG, PLEX, MMF | Death from MG complications | NR |
| 36/75/M | NSCLC | No | Nivolumab | 4 | Ptosis, diplopia, nasal speech/weakness of the palatal muscles, limb/neck weakness, myalgia, dyspnea (MGFA IV) | -/NR | + (12119) | +/- | GC, IVIG, tacrolimus | Improvement | NR |
| 37/74/F | CRC | No | Nivolumab | 5.3 | Ptosis, dysphagia, limb/neck weakness, myalgia, dyspnea (MGFA V) | +/NR | + (5331) | +/- | GC, pyridostigmine, IVIG, PLEX, NIPPV | Improvement | NR |
| 38/80/M | Melanoma | No | Nivolumab | 2.42 | Ptosis, limb weakness, dyspnea (MGFA V) | +/NR | + (7740) | +/+ | GC, IA, IVIG, PLEX, ETI | Improvement | SD |
| 39/81/F | Melanoma | No | Nivolumab | 3 | Ptosis, diplopia, limb weakness, myalgia, dyspnea[^c^](https://en.wikipedia.org/wiki/Vertical_Bar) (MGFA V) | +/NR | + (8729) | -/- | GC | Death from MG complications | NR |
| 40/73/M | Melanoma | No | Nivolumab | 11.14 | Ptosis, blurry vision, dyspnea (MGFA V) | +/+ | + (559) | +/- | GC, PLEX, IVIG, NIPPV | Complete resolution | PD |
| 41/78/M | Melanoma | No | Pembrolizumab^d^ | 4.5 | Ptosis, limb/facial weakness, dyspnea (MGFA V) | +/- | + (2835) | +/+ | GC, pyridostigmine, IVIG, tacrolimus, NIPPV | Death from MG complications^b^ | PR |
| 42/75/M | Melanoma | Yes | Pembrolizumab | 3 | Ptosis, diplopia, dysphagia, dyspnea (MGFA V) | +/NR | + (594) | -/- | GC, pyridostigmine, IVIG, PLEX, MMF, rituximab, NIPPV | Improvement | PR |
| 43/75/M | Melanoma | Yes | Pembrolizumab | 3.57 | Ptosis, limb/neck weakness dyspnea (MGFA V) | +/NR | - (NR) | -/- | GC, IVIG, NIPPV | Complete resolution | SD |
| 44/84/M | Melanoma | No | Pembrolizumab | 4 | Ptosis, dysphagia, limb/neck weakness, dyspnea (MGFA V) | +/NR | - (NR) | -/- | GC, pyridostigmine, IVIG, NIPPV | Improvement | PR |
| 45/63/M | Melanoma | No | Pembrolizumab | 2.00 | Ptosis, blurry vision, facial weakness, dyspnea (MGFA V) | +/NR | + (10386) | +/- | GC, pyridostigmine, IVIG, PLEX, ETI | Death from MG complications | NR |
| 46/86/M | Melanoma | No | Pembrolizumab | 4.7 | Dysphagia, dysarthria, limb/neck weakness, dyspnea (MGFA V) | +/+ | + (444) | +/- | GC, PLEX, ETI | Death from MG complications | NR |
| 47/73/M | Melanoma | Yes | Pembrolizumab | 3.85 | Dyspnea (MGFA V) | ND/ND | ND | -/- | GC, pyridostigmine, MMF, NIPPV | Complete resolution | PD |
| 48/75/F | Mesothelioma | No | Pembrolizumab | 2 | Ptosis, dysphagia, limb/neck weakness, dyspnea^c^ (MGFA V) | +/NR | + (2970) | -/- | GC, IVIG, PEG, NIPPV | Death from MG complications | NR |
| 49/67/F | NSCLC | Yes | Atezolizumab | 6.00 | Dysphagia, limb/neck weakness, dyspnea (MGFA V) | ND/NR | ND | -/- | GC, pyridostigmine, PLEX, NIPPV | Improvement | NR |
| 50/69/F | NSCLC | No | Nivolumab | 5 | Diplopia, dyspnea (MGFA V) | +/NR | + (1156) | -/+ | GC, NIPPV, pacemaker | Improvement | NR |
| 51/76/F | NSCLC | No | Nivolumab | 3.71 | Ptosis, diplopia, dysphagia, limb/neck weakness, myalgia, dyspnea (MGFA V) | +/NR | + (6566) | -/- | GC, IA, IVIG, PLEX, NIPPV | Improvement | PD |
| 52/68/F | NSCLC | Yes (Ocular) | Nivolumab | 10.00 | Diplopia, dysphagia, dysarthria, nasal speech/weakness of the palatal muscles, limb/facial weakness, dyspnea^c^ (MGFA V) | +/NR | NR | -/- | GC, pyridostigmine, PLEX, NIPPV | Deterioration | PR |
| 53/45/M | NSCLC | No | Nivolumab | 2.00 | Ptosis, dyspnea (MGFA V) | +/NR | + (NR) | +/- | GC, pyridostigmine, IVIG, ETI | Improvement | PR |
| 54/65/M | NSCLC | No | Nivolumab | 5 | Ptosis, diplopia, dysphagia, limb/neck weakness, dyspnea^c^ (MGFA V) | -/NR | + (2216) | +/- | GC, pyridostigmine | Death from MG complications | NR |
| 55/68/M | NSCLC | No | Nivolumab | 4.14 | Ptosis, diplopia, dysphagia, limb/neck weakness, dyspnea (MGFA V) | +/NR | + (9892) | -/+ | GC, IVIG, PLEX, IABP, ETI | Death from MG complications | NR |
| 56/65/M | Renal cancer | No | Nivolumab^e^ | 4 | Ptosis, diplopia, blurry vision, dysphonia, nasal speech, limb/neck weakness, myalgia, incontinence, dyspnea, (MGFA V) | -/+ | + (2299) | +/- | GC, pyridostigmine, PLEX, IVIG, tacrolimus, ETI | Death from MG complications | Not assessed |
| 57/75/M | Renal cancer | No | Nivolumab | 3.57 | Ptosis, diplopia, dysphagia, generalized weakness, dyspnea (MGFA V) | +/NR | + (1587) | -/- | Pyridostigmine, IVIG, NIPPV | Improvement | NR |
| 58/73/M | Renal cancer | No | Nivolumab | 2.57 | Limb weakness, myalgia, dyspnea (MGFA V) | +/NR | + (8950) | -/- | GC, pyridostigmine, IVIG, PLEX, ETI | Deterioration | PD |
| 59/67/M | Renal cancer | No | Nivolumab | 7 | Diplopia, dysphagia, dysarthria, generalized weakness, incontinence, dyspnea (MGFA V) | +/NR | + (418) | -/- | GC, IVIG, PLEX, rituximab, PEG, ETI | Improvement | NR |
| 60/65/M | Renal cancer | No | Nivolumab | 2.14 | Ptosis, diplopia, limb/neck weakness, myalgia, dyspnea^c^ (MGFA V) | +/- | + (6321) | +/- | GC, IVIG | Death from MG complications | NR |
| 61/65/F | Sarcoma | No | Durvalumab/Tremelimumab | 3 | Ptosis, dysphagia, dysarthria, limb/facial/neck weakness, myalgia, dyspnea (MGFA V) | +/+ | + (19794) | -/- | GC, PLEX, IVIG, NIPPV | Improvement | PD |
| 62/70/M | SCLC | No | Ipilimumab/Nivolumab | 2.28 | Ptosis, diplopia, generalized weakness, dyspnea (MGFA V) | +/+ | - (NR) | -/- | GC, IVIG, PLEX, ETI | Death from MG complications | NR |
| 63/57/M | Thymoma | Yes | Pembrolizumab | 1.42 | Ptosis, dyspnea (MGFA V) | NR/NR | + (8226) | -/- | GC, pyridostigmine, PLEX, PEG, ETI | Death from MG complications | NR |
| 64/81/F | Urachal adenocarcinoma | No | Atezolizumab | 16 | Ptosis, dysphagia, dysarthria, dysphonia, generalized weakness, limb/facial/neck weakness, dyspnea (MGFA V) | -/- | + (554) | +/- | GC, pyridostigmine, PLEX, MMF, rituximab, ETI | Death from MG complications | Not assessed |
| 65/83/M | Urothelial carcinoma | No | Nivolumab^f^ | 3.42 | Dysphagia, dysphonia, limb weakness, dyspnea (MGFA V) | +/+ | + (4135) | +/- | GC, pyridostigmine, PLEX, IVIG, MMF, rituximab, tacrolimus, PEG, ETI | Improvement | PD |

^a^Abbreviations, MG, myasthenia gravis; ICI, immune checkpoint inhibitor; MGFA, Myasthenia Gravis Foundation of America; AChR, acetylcholine receptor; CPK, creatine kinase; M, male; N/A, not applicable; -, absent or within normal levels; +, present or elevated levels; NR, not reported; F, female; GC, glucocorticoids; SD, stable disease; ND, not done; PR, partial response; IVIG, intravenous immunoglobulin; CR, complete response; NSCLC, non-small cell lung cancer; PLEX, plasmapheresis; PD, progressive disease; MMF, mycophenolic acid; SCC, squamous cell carcinoma; PEG, percutaneous endoscopic gastrostomy; NIPPV, non-invasive positive pressure ventilation; ETI, endotracheal intubation.

^b^The patient had gradual improvement in all symptoms but then died from aspiration pneumonia

^c^The patient developed respiratory failure but refused endotracheal intubation

^d^The patient also received NKTR

^e^The patient also received sitravatinib

^f^The patient also received azacitadine
