# Supplementary material for: Immune checkpoint inhibitor related myasthenia gravis: single center experience and systematic review of the literature
Source: J Immunother Cancer. 2019 Nov 21;7:319. doi: 10.1186/s40425-019-0774-y (PMC6868691; doi:10.1186/s40425-019-0774-y)
Supplement: Supplementary file 5 — Additional file 5: Table S3. Quality Appraisal of the Literature Reported Cases. [file 40425_2019_774_MOESM5_ESM.docx]

**Table S3.** Quality Appraisal of the Literature Reported Cases.

| **Lead author (citation)** | **Title** | **Patient demographics** | **Current health status** | **Medical history** | **Physical exam** | **Patient disposition** | **Drug identification** | **Dosage** | **Drug reaction interface** | **Concomitant therapy** | **Adverse events** | **Discussion** |
| --- | --- | --- | --- | --- | --- | --- | --- | --- | --- | --- | --- | --- |
| Alnahhas et al(1) | Agree | Agree | Agree | Agree | Agree | Agree | Agree | Agree | Agree | Agree | Agree | Agree |
| Benn et al(2) | Agree | Agree | Agree | Agree | Agree | Agree | Agree | Agree | Agree | Agree | Agree | Agree |
| Chae et al(3) | Agree | Agree | Agree | Agree | Agree | Agree | Agree | Partially agree | Agree | Agree | Agree | Partially agree |
| Chang et al(4) | Agree | Agree | Agree | Agree | Agree | Agree | Agree | Partially agree | Agree | Agree | Agree | Agree |
| Chen et al(5) | Agree | Agree | Agree | Agree | Agree | Agree | Agree | Agree | Agree | Agree | Agree | Agree |
| Chen et al(6) | Agree | Agree | Agree | Agree | Agree | Agree | Agree | Agree | Agree | Agree | Agree | Agree |
| Cooper et al(7) | Agree | Agree | Agree | Agree | Agree | Agree | Agree | Agree | Agree | Agree | Agree | Agree |
| De Chabot et al(8) | Agree | Agree | Agree | Agree | Agree | Agree | Agree | Partially agree | Agree | Agree | Agree | Agree |
| Earl et al(9) | Agree | Agree | Agree | Agree | Agree | Agree | Agree | Partially agree | Agree | Agree | Agree | Agree |
| Fellner et al(10) | Agree | Agree | Agree | Agree | Agree | Agree | Agree | Partially agree | Agree | Agree | Agree | Agree |
| Fukasawa et al(11) | Agree | Agree | Agree | Agree | Agree | Agree | Agree | Agree | Agree | Agree | Agree | Agree |
| Gonzalez et al(12) | Agree | Agree | Agree | Agree | Agree | Agree | Agree | Partially agree | Agree | Agree | Agree | Agree |
| Habib et al(13) | Agree | Agree | Agree | Agree | Agree | Agree | Agree | Partially agree | Agree | Agree | Agree | Agree |
| Hasegawa et al(14) | Agree | Agree | Agree | Agree | Agree | Agree | Agree | Agree | Agree | Agree | Agree | Agree |
| Hibino et al(15) | Agree | Agree | Agree | Agree | Agree | Agree | Agree | Agree | Agree | Agree | Agree | Agree |
| Huh et al(16) | Agree | Agree | Agree | Agree | Agree | Agree | Agree | Agree | Agree | Agree | Agree | Agree |
| Johnson et al(17) | Agree | Agree | Agree | Agree | Agree | Agree | Agree | Agree | Agree | Agree | Agree | Agree |
| Kamada et al(18) | Agree | Agree | Agree | Agree | Agree | Agree | Agree | Partially agree | Agree | Agree | Agree | Agree |
| Kang et al(19) | Agree | Agree | Agree | Agree | Agree | Agree | Agree | Agree | Agree | Agree | Agree | Agree |
| Kimura et al(20) | Agree | Agree | Agree | Agree | Agree | Agree | Agree | Agree | Agree | Agree | Agree | Agree |
| Kolb et al(21) | Agree | Agree | Agree | Agree | Agree | Agree | Agree | Partially agree | Agree | Agree | Agree | Agree |
| Konoeda et al(22) | Agree | Agree | Agree | Agree | Agree | Agree | Agree | Agree | Agree | Agree | Agree | Agree |
| Lau et al(23) | Agree | Agree | Agree | Agree | Agree | Agree | Agree | Partially agree | Agree | Agree | Agree | Agree |
| Liao et al(24) | Agree | Agree | Agree | Agree | Agree | Agree | Agree | Partially agree | Agree | Agree | Agree | Agree |
| Liewluck et al(25) | Agree | Agree | Agree | Agree | Agree | Agree | Agree | Agree | Agree | Agree | Agree | Agree |
| Loochtan et al(26) | Agree | Agree | Agree | Agree | Agree | Agree | Agree | Partially agree | Agree | Agree | Agree | Agree |
| Lopez et al(27) | Agree | Agree | Agree | Agree | Agree | Agree | Agree | Agree | Agree | Agree | Agree | Agree |
| Maeda et al(28) | Agree | Agree | Agree | Agree | Agree | Agree | Agree | Agree | Agree | Agree | Agree | Agree |
| Makarious et al(29) | Agree | Agree | Agree | Agree | Agree | Agree | Agree | Agree | Agree | Agree | Agree | Agree |
| March et al(30) | Agree | Agree | Agree | Agree | Agree | Agree | Agree | Partially agree | Agree | Agree | Agree | Agree |
| Mehta et al(31) | Agree | Agree | Agree | Agree | Agree | Agree | Agree | Partially agree | Agree | Agree | Agree | Agree |
| Mitsune et al(32) | Agree | Agree | Agree | Agree | Agree | Agree | Agree | Agree | Agree | Agree | Agree | Agree |
| Montes et al(33) | Agree | Agree | Agree | Agree | Agree | Agree | Agree | Agree | Agree | Agree | Agree | Agree |
| Nguyen et al(34) | Agree | Agree | Agree | Agree | Agree | Agree | Agree | Agree | Agree | Agree | Agree | Agree |
| Phadke et al(35) | Agree | Agree | Agree | Agree | Agree | Agree | Agree | Agree | Agree | Agree | Agree | Agree |
| Polat et al(36) | Agree | Agree | Agree | Agree | Agree | Agree | Agree | Agree | Agree | Agree | Agree | Agree |
| Rutzen et al(37) | Agree | Agree | Agree | Agree | Agree | Agree | Agree | Partially agree | Agree | Agree | Agree | Agree |
| Sakai et al(38) | Agree | Agree | Agree | Agree | Agree | Partially agree | Agree | Partially agree | Agree | Agree | Agree | Agree |
| Sciacca et al(39) | Agree | Agree | Agree | Agree | Agree | Agree | Agree | Agree | Agree | Agree | Agree | Agree |
| Shah et al(40) | Agree | Agree | Agree | Agree | Agree | Agree | Agree | Agree | Agree | Agree | Agree | Agree |
| Shirai et al(41) | Agree | Agree | Agree | Agree | Agree | Agree | Agree | Agree | Agree | Agree | Agree | Agree |
| Suzuki et al(42) | Agree | Agree | Agree | Agree | Agree | Agree | Agree | Agree | Agree | Partially agree | Agree | Agree |
| Tan et al(43) | Agree | Agree | Agree | Agree | Agree | Partially agree | Agree | Partially agree | Agree | Agree | Agree | Agree |
| Tanaka et al(44) | Agree | Agree | Agree | Agree | Agree | Agree | Agree | Agree | Agree | Agree | Agree | Agree |
| Zhu et al(45) | Agree | Agree | Agree | Agree | Agree | Agree | Agree | Agree | Agree | Partially agree | Agree | Agree |
| Zimmer et al(46) | Agree | Agree | Agree | Agree | Agree | Partially agree | Agree | Partially agree | Agree | Agree | Agree | Agree |

To evaluate the quality of the case reports retrieved from the literature, we used the guidelines recommended by the International Society for Pharmacoepidemiology and the International Society of Pharmacovigilance for publishing adverse events reports(47). The assessment was carried out by one investigators and cross-checked by another investigators. We only used the items reported by the guidelines as required information, without regard to the items reported as desirable or relevant. The items appraised included: i) relevance of the title to the reported information, ii) adequate description of the patient (demographics, existing health condition, relevant past medical history, physical and laboratory abnormalities, and significant morbidity or mortality), iii) adequate description of the drug (identification of generic and trade names of the drug and the manufacturer, drug dosage, duration between drug administration and adverse events, and concomitant therapy that could potentially contributes to occurrence of adverse events), iv) adequate description of the adverse events and their outcome, and v) discussion of the evidence supporting the causal association between the drug and the adverse events. Possible item ratings are yes, partially, or no. Disagreement was resolved by discussion until consensus was reached.

REFERENCES

1. Alnahhas I, Wong J. A case of new-onset antibody-positive myasthenia gravis in a patient treated with pembrolizumab for melanoma. Muscle Nerve. 2017;55(6):E25-E6.

2. Benn BS, Lombard CM, Krishna G. Nivolumab-Induced Granulomatous Inflammation of the Pleura. J Thorac Oncol. 2017;12(7):e100-e1.

3. Chae J, Peikert T. Myasthenia Gravis Crisis Complicating Anti-PD-L1 Cancer Immunotherapy. Critical Care Medicine. 2017;46(1):280.

4. Chang E, Sabichi AL, Sada YH. Myasthenia Gravis After Nivolumab Therapy for Squamous Cell Carcinoma of the Bladder. J Immunother. 2017;40(3):114-6.

5. Chen JH, Lee KY, Hu CJ, Chung CC. Coexisting myasthenia gravis, myositis, and polyneuropathy induced by ipilimumab and nivolumab in a patient with non-small-cell lung cancer: A case report and literature review. Medicine (Baltimore). 2017;96(50):e9262.

6. Chen YH, Liu FC, Hsu CH, Chian CF. Nivolumab-induced myasthenia gravis in a patient with squamous cell lung carcinoma: Case report. Medicine (Baltimore). 2017;96(27):e7350.

7. Cooper DS, Meriggioli MN, Bonomi PD, Malik R. Severe Exacerbation of Myasthenia Gravis Associated with Checkpoint Inhibitor Immunotherapy. J Neuromuscul Dis. 2017;4(2):169-73.

8. de Chabot G, Justeau G, Pinquie F, Nadaj-Pakleza A, Hoppe E, Hureaux J, et al. [Unexpected adverse events of immunotherapies in non-small cell lung cancer: About 2 cases]. Rev Pneumol Clin. 2017;73(6):326-30.

9. Earl DE, Loochtan AI, Bedlack RS. Refractory myasthenia gravis exacerbation triggered By pembrolizumab. Muscle Nerve. 2018;57(4):E120-E1.

10. Fellner A, Makranz C, Lotem M, Bokstein F, Taliansky A, Rosenberg S, et al. Neurologic complications of immune checkpoint inhibitors. J Neurooncol. 2018;137(3):601-9.

11. Fukasawa Y, Sasaki K, Natsume M, Nakashima M, Ota S, Watanabe K, et al. Nivolumab-Induced Myocarditis Concomitant with Myasthenia Gravis. Case Rep Oncol. 2017;10(3):809-12.

12. Gonzalez NL, Puwanant A, Lu A, Marks SM, Zivkovic SA. Myasthenia triggered by immune checkpoint inhibitors: New case and literature review. Neuromuscul Disord. 2017;27(3):266-8.

13. Habib R, Gordon J, Zielinski R. A Cascade of Pembrolizumab-Induced Autoimmune Toxicities with Mixed Responses to Corticosteroids. J Thorac Oncol. 2018;13(1):e8-e9.

14. Hasegawa Y, Kawai S, Ota T, Tsukuda H, Fukuoka M. Myasthenia gravis induced by nivolumab in patients with non-small-cell lung cancer: a case report and literature review. Immunotherapy. 2017;9(9):701-7.

15. Hibino M, Maeda K, Horiuchi S, Fukuda M, Kondo T. Pembrolizumab-induced myasthenia gravis with myositis in a patient with lung cancer. Respirol Case Rep. 2018;6(7):e00355.

16. Huh SY, Shin SH, Kim MK, Lee SY, Son KH, Shin HY. Emergence of Myasthenia Gravis with Myositis in a Patient Treated with Pembrolizumab for Thymic Cancer. J Clin Neurol. 2018;14(1):115-7.

17. Johnson DB, Saranga-Perry V, Lavin PJ, Burnette WB, Clark SW, Uskavitch DR, et al. Myasthenia Gravis Induced by Ipilimumab in Patients With Metastatic Melanoma. J Clin Oncol. 2015;33(33):e122-4.

18. Kamada S, Hanazono A, Sanpei Y, Fukunaga H, Inoue T, Suzuki S, et al. Anti-titin antibody, one of the antistriational autoantibodies was found in a case of seronegative myasthenia gravis associated with anti-PD-1 therapy. Journal of the Neurological Sciences. 2017;381:1145.

19. Kang KH, Grubb W, Sawlani K, Gibson MK, Hoimes CJ, Rogers LR, et al. Immune checkpoint-mediated myositis and myasthenia gravis: A case report and review of evaluation and management. Am J Otolaryngol. 2018;39(5):642-5.

20. Kimura T, Fukushima S, Miyashita A, Aoi J, Jinnin M, Kosaka T, et al. Myasthenic crisis and polymyositis induced by one dose of nivolumab. Cancer Sci. 2016;107(7):1055-8.

21. Kolb NA, Trevino CR, Waheed W, Sobhani F, Landry KK, Thomas AA, et al. Neuromuscular complications of immune checkpoint inhibitor therapy. Muscle Nerve. 2018.

22. Konoeda F, Suzuki S, Nishimoto Y, Hoshino H, Takagi M. A case of myasthenia gravis and myositis induced by nivolumab. Rinsho Shinkeigaku. 2017;57(7):373-7.

23. Lau KH, Kumar A, Yang IH, Nowak RJ. Exacerbation of myasthenia gravis in a patient with melanoma treated with pembrolizumab. Muscle Nerve. 2016;54(1):157-61.

24. Liao B, Shroff S, Kamiya-Matsuoka C, Tummala S. Atypical neurological complications of ipilimumab therapy in patients with metastatic melanoma. Neuro Oncol. 2014;16(4):589-93.

25. Liewluck T, Kao JC, Mauermann ML. PD-1 Inhibitor-associated Myopathies: Emerging Immune-mediated Myopathies. J Immunother. 2018;41(4):208-11.

26. Loochtan AI, Nickolich MS, Hobson-Webb LD. Myasthenia gravis associated with ipilimumab and nivolumab in the treatment of small cell lung cancer. Muscle Nerve. 2015;52(2):307-8.

27. Lopez D, Calvo A, Fershko A. Myasthenia Gravis and Rhabdomyolysis in A Patient with Advanced Renal Cell Cancer Treated With Nivolumab: A Case Report And Review of the Literature. British Journal of Medical and Health Research. 2015;2(12).

28. Maeda O, Yokota K, Atsuta N, Katsuno M, Akiyama M, Ando Y. Nivolumab for the treatment of malignant melanoma in a patient with pre-existing myasthenia gravis. Nagoya J Med Sci. 2016;78(1):119-22.

29. Makarious D, Horwood K, Coward JIG. Myasthenia gravis: An emerging toxicity of immune checkpoint inhibitors. Eur J Cancer. 2017;82:128-36.

30. March KL, Samarin MJ, Sodhi A, Owens RE. Pembrolizumab-induced myasthenia gravis: A fatal case report. J Oncol Pharm Pract. 2018;24(2):146-9.

31. Mehta JJ, Maloney E, Srinivasan S, Seitz P, Cannon M. Myasthenia Gravis Induced by Nivolumab: A Case Report. Cureus. 2017;9(9):e1702.

32. Mitsune A, Yanagisawa S, Fukuhara T, Miyauchi E, Morita M, Ono M, et al. Relapsed Myasthenia Gravis after Nivolumab Treatment. Intern Med. 2018;57(13):1893-7.

33. Montes V, Sousa S, Pita F, Guerreiro R, Carmona C. Myasthenia Gravis Induced by Ipilimumab in a Patient With Metastatic Melanoma. Front Neurol. 2018;9:150.

34. Nguyen BH, Kuo J, Budiman A, Christie H, Ali S. Two cases of clinical myasthenia gravis associated with pembrolizumab use in responding melanoma patients. Melanoma Res. 2017;27(2):152-4.

35. Phadke SD, Ghabour R, Swick BL, Swenson A, Milhem M, Zakharia Y. Pembrolizumab Therapy Triggering an Exacerbation of Preexisting Autoimmune Disease: A Report of 2 Patient Cases. J Investig Med High Impact Case Rep. 2016;4(4):2324709616674316.

36. Polat P, Donofrio PD. Myasthenia gravis induced by nivolumab therapy in a patient with non-small-cell lung cancer. Muscle Nerve. 2016;54(3):507.

37. Rutzen-Lopez IM, Fu JB, Arias-Berrios JE. Nivolumab-Induced Concurrent Guillain-Barre Syndrome and Myasthenia Gravis in a Patient with Metastatic Renal Cell Carcinoma: A Case Report. PM&R 2017;9(9):s246-s7.

38. Sakai S, Tajiri K, Hasegawa J, Hashimoto N, Yomamoto YS, Seo Y, et al. Nivolumab-induced Autoimmune Myocarditis: A Case Report. Journal of Cardiac Failure. 2017;23(10):32.

39. Sciacca G, Nicoletti A, Rampello L, Noto L, Parra HJ, Zappia M. Benign form of myasthenia gravis after nivolumab treatment. Muscle Nerve. 2016;54(3):507-9.

40. Shah M, Tayar JH, Abdel-Wahab N, Suarez-Almazor ME. Myositis as an adverse event of immune checkpoint blockade for cancer therapy. Semin Arthritis Rheum. 2019;48(4):736-40.

41. Shirai T, Sano T, Kamijo F, Saito N, Miyake T, Kodaira M, et al. Acetylcholine receptor binding antibody-associated myasthenia gravis and rhabdomyolysis induced by nivolumab in a patient with melanoma. Jpn J Clin Oncol. 2016;46(1):86-8.

42. Suzuki S, Ishikawa N, Konoeda F, Seki N, Fukushima S, Takahashi K, et al. Nivolumab-related myasthenia gravis with myositis and myocarditis in Japan. Neurology. 2017;89(11):1127-34.

43. Tan RYC, Toh CK, Takano A. Continued Response to One Dose of Nivolumab Complicated by Myasthenic Crisis and Myositis. J Thorac Oncol. 2017;12(7):e90-e1.

44. Tanaka R, Sunada Y, Wataru F. Successful reinstitution of nivolumab in combination with corticosteroids for metastatic malignant melanoma with myasthenia gravis as an immune-related adverse event. Kawasaki Medical Journal. 2017;43(2):59-61.

45. Zhu J, Li Y. Myasthenia gravis exacerbation associated with pembrolizumab. Muscle Nerve. 2016;54(3):506-7.

46. Zimmer L, Goldinger SM, Hofmann L, Loquai C, Ugurel S, Thomas I, et al. Neurological, respiratory, musculoskeletal, cardiac and ocular side-effects of anti-PD-1 therapy. Eur J Cancer. 2016;60:210-25.

47. Kelly WN, Arellano FM, Barnes J, Bergman U, Edwards IR, Fernandez AM, et al. Guidelines for submitting adverse event reports for publication. Pharmacoepidemiol Drug Saf. 2007;16(5):581-7.
