# Supplementary material for: Immune checkpoint inhibitor related myasthenia gravis: single center experience and systematic review of the literature
Source: J Immunother Cancer. 2019 Nov 21;7:319. doi: 10.1186/s40425-019-0774-y (PMC6868691; doi:10.1186/s40425-019-0774-y)
Supplement: Supplementary file 6 — Additional file 6: Table S4. Diagnostic tools used in patients with suspected ICI-related MG. [file 40425_2019_774_MOESM6_ESM.docx]

**Table S4.** Diagnostic tools used in patients with suspected ICI-related MG.^a^

| **Variable** | **No. (%)** |
| --- | --- |
| Diagnostic tools (no. tested) |  |
| Auto antibody panel positive titers |  |
| Anti-AChR (*n=*56) | 37 (66) |
| Anti-Striated muscle (*n=*18) | 12 (67) |
| ANA (*n=*10) | 2 (20) |
| Anti-titin (*n=*2) | 1 (50) |
| Anti-MuSK (*n=*35) | 1 (3) |
| Voltage-gated calcium channel (*n=*15) | 0 |
| Antiaminoacyl tRNA synthetase (*n=*7) | 0 |
| Muscle enzymes elevation |  |
| CPK (*n=*49) | 41 (84) |
| Troponin (*n=*14) | 13 (93) |
| CKMB (*n=*8) | 8 (100) |
| Aldolase (*n=*6) | 5 (83) |
| Myoglobin (*n=*4) | 3 (75) |
| Transaminases elevation (ALT, AST) (*n=*25) | 21 (84) |
| Edrophonium test positive (*n=*5)^b^ | 4 (80) |
| Ice pack test positive (*n=*4)^b^ | 2 (50) |
| Electrodiagnostic studies (skeletal muscle EMG, RNS, NCS) (*n=*37) |  |
| MG^c^  Myopathy^d^  MG and myopathy^d^  Polyneuropathy  No pathologic findings | 16 (43)  6 (16)  6 (16)  3 (8)  6 (16) |
| Imaging of the Neck (MRI, CT, X-ray) (*n=*29)  Thymoma | 0 |
| Imaging of the brain (MRI, CT) (*n=*27)  Acute intracranial events | 0 |
| MRI of the heart (*n=*2)  Myocarditis | 0 |
| TTE (*n=*15) |  |
| Left ventricular dysfunction | 4 (27) |
| EKG abnormalities (*n=*15)  Diffuse ST elevation  Premature ventricular contraction  Ventricular tachycardia | 2 (13)  1 (7)  1 (7) |
| RBBB | 1 (7) |
| Coronarography (*n=*2) |  |
| Arterial stenosis | 0 |
| Skeletal muscle biopsy (*n=*7)  Inflammatory infiltrates  Necrotizing myopathy  Inconclusive^e^  Myocardial biopsy (*n=*3)  Inflammatory infiltrates | 5 (71)  1 (14)  1 (14)  3 (100) |

^a^Abbreviations: Anti-AChR, Anti-Acetylcholine receptor; ANA, Anti-nuclear antibody; Anti-MuSK, Anti-muscle specific kinase; CPK, creatine phosphokinase; CKMB, creatine kinase MB; ALT, alanine transaminase; AST, aspartate transaminase; EMG, electromyography; RNS, repetitive nerve stimulation; NCS, nerve conduction study; MG, myasthenia gravis; MRI, magnetic resonance imaging; CT, computed tomography; TTE, transthoracic echocardiography; EKG, electrocardiogram; RBBB, right bundle branch block. Numbers are rounded to the nearest whole number.

^b^One patient had a partially positive test result.

^c^Three patients also had findings suggestive of polyneuropathy.

^d^Two patients also had findings suggestive of polyneuropathy.

^e^The patient also had a nerve biopsy which was inconclusive.
