# Supplementary material for: Immune checkpoint inhibitor related myasthenia gravis: single center experience and systematic review of the literature
Source: J Immunother Cancer. 2019 Nov 21;7:319. doi: 10.1186/s40425-019-0774-y (PMC6868691; doi:10.1186/s40425-019-0774-y)
Supplement: Supplementary file 7 — Additional file 7: Table S5. MG outcomes and survival data for patients who were tested for muscle enzymes elevation and those who were not. [file 40425_2019_774_MOESM7_ESM.docx]

**Table S5**. MG outcomes and survival data for patients who were tested for muscle enzymes elevation (*n=*49) and those who were not (*n=*16).^a^

| Variable | Muscle enzymes tested; *n (%)* | | | Muscle enzymes not tested (*n=16); n (%)* |
| --- | --- | --- | --- | --- |
|  | CPK/Troponin levels elevated (*n=*41) | CPK/Troponin levels normal (*n=*8) | **Total (*n=*49)**^b^ |  |
| MG outcome |  |  |  |  |
| Complete resolution | 5/40^c^ (13) | 3 (38) | 8 (17) | 4/14^d^ (25) |
| Improvement | 22/40^c^ (55) | 4 (50) | 26 (54) | 8/14^d^ (50) |
| Deterioration | 13/40^c^ (33) | 1 (13) | 14 (29) | 2/14^d^ (13) |
| Death |  |  |  |  |
| MG complication | 13 (32) | 1 (13) | 14 (29) | 1 (6) |
| Cancer | 3 (7) | 0 | 3 (6) | 1 (6) |
| Other comorbidities | 1 (2) | 2 (25) | 3 (6) | 0 |
| Inconclusive | 2 (5) | 0 | 2 (4) | 0 |

^a^Abbreviations: CPK, creatine phosphokinase; MG, myasthenia gravis. Numbers are rounded to the nearest whole number.

^b^Forty nine patients were tested for CPK including 14 who were also tested for troponin.

^c^Data were not reported for one patient.

^d^Two patients with pre-existing MG did not develop a flare of their disease after ICI initiation and were excluded from the analysis.
