# Supplementary material for: Immune checkpoint inhibitor related myasthenia gravis: single center experience and systematic review of the literature
Source: J Immunother Cancer. 2019 Nov 21;7:319. doi: 10.1186/s40425-019-0774-y (PMC6868691; doi:10.1186/s40425-019-0774-y)
Supplement: Supplementary file 8 — Additional file 8: Table S6. Clinical characteristics, diagnostic findings, management, and outcomes of ICI-related MG in the whole cohort and in patients with a definite diagnosis of MG. [file 40425_2019_774_MOESM8_ESM.docx]

**Table S6.** Clinical characteristics, diagnostic findings, management, and outcomes of ICI-related MG in the whole cohort (*n=*65) and in patients with a definite diagnosis of MG (*n=*58).^a^

| **Variable** | **Total Cohort (*n=*65), *n* (%)** | **Definite MG (*n*=58); *n* (%)** |
| --- | --- | --- |
| MGFA classification |  |  |
| I | 8 (12) | 6 (10) |
| II | 14 (22) | 11 (19) |
| III | 8 (12) | 8 (14) |
| IV | 4 (6) | 3 (5) |
| V | 29 (45) | 28 (48) |
| Clinical presentation |  |  |
| Ptosis | 49 (75) | 42 (72) |
| Dyspnea | 40 (62) | 36 (62) |
| Limb weakness | 36 (55) | 34 (59) |
| Dysphagia | 31 (48) | 27 (47) |
| Diplopia | 27 (42) | 24 (41) |
| Neck weakness | 22 (34) | 20 (35) |
| Myalgias | 13 (20) | 11 (19) |
| Blurry vision | 7 (11) | 6 (10) |
| Dysarthria | 8 (12) | 7 (12) |
| Generalized weakness | 6 (9) | 4 (7) |
| Dysphonia | 7 (11) | 7 (12) |
| Facial weakness | 8 (12) | 8 (14) |
| Nasal speech/weakness of the palatal muscles | 6 (9) | 6 (10) |
| Incontinence | 2 (3) | 2 (3) |
| Diagnostic tools |  |  |
| Auto antibody panel positive titers |  |  |
| Anti-AChR | 37/56 (66) | 37/51 (73) |
| Anti-Striated muscle | 12/18 (67) | 11/17 (65) |
| Muscle enzymes elevation |  |  |
| CPK | 41/49 (84) | 37/44 (84) |
| Troponin | 13/14 (93) | 11/12 (92) |
| Edrophonium test positive | 4/5 (80) | 4/5 (80) |
| Ice pack test positive | 2/4 (50) | 2/4 (50) |
| Electrodiagnostic studies (skeletal muscle EMG, RNS, NCS) |  |  |
| MG  Myopathy  MG and myopathy  Polyneuropathy  No pathologic findings | 16/37 (43)  6/37 (16)  6/37 (16)  3/37 (8)  6/37 (16) | 16/34 (47)  5/34 (15)  6/34 (18)  2/34 (6)  5/34 (15) |
| Treatment of MG^b^ |  |  |
| Corticosteroids | 59/63 (94) | 52/56 (93) |
| Acetylcholinesterase inhibitors | 32/63 (51) | 28/56 (50) |
| IVIG | 30/63 (48) | 28/56 (50) |
| Plasmapheresis | 28/63 (44) | 26/56 (46) |
| Other IST (MMF, rituximab, infliximab or tacrolimus) | 10/63 (16) | 10/56 (18) |
| IA | 1/63 (2) | 3/56 (5) |
| ICI holding/discontinuation | 61/63 (97) | 54/56 (96) |
| MG outcome^b,c^ |  |  |
| Complete resolution | 12/62 (19) | 10/56 (18) |
| Improvement | 34/62 (55) | 32/56 (57) |
| Deterioration | 16/62 (26) | 14/56 (25) |
| Death | 24 (37) | 21 (36) |
| MG complications | 15 (23) | 13 (22) |
| Cancer progression | 4 (6) | 4 (7) |
| Other comorbidities | 3 (5) | 2 (3) |
| Unspecified | 2 (3) | 2 (3) |

^a^Abbreviations: MG, myasthenia gravis; MGFA, Myasthenia Gravis Foundation of America; Anti-AChR, Anti-Acetylcholine receptor; CPK, creatine phosphokinase; EMG, electromyography; RNS, repetitive nerve stimulation; NCS, nerve conduction study; IVIG, intravenous immunoglobulin; IST, immunosuppressive therapy; MMF, mycophenolic acid; IA, immunoadsorption; ICI, immune checkpoint inhibitors. Numbers are rounded to the nearest whole number. Numbers are rounded to the nearest whole number.

^b^Two patients with pre-existing MG did not develop a flare of their disease after ICI initiation and were excluded from the analysis.

^c^Data were not reported for one patient.
